# Mastering your fellowship: Part 4, 2025

**DOI:** 10.4102/safp.v67i1.6144

**Published:** 2025-04-15

**Authors:** Mergan Naidoo, Klaus von Pressentin, John M. Musonda, Selvandran Rangiah, Michele Torlutter, Joyce S. Musonda

**Affiliations:** 1 Department of Family Medicine, College of Health Science, University of KwaZulu-Natal, Durban, South Africa; 2 Department of Family, Community and Emergency Care, Faculty of Health Sciences, University of Cape Town, Cape Town, South Africa; 3 Department of Family Medicine, Faculty of Medicine, University of the Witwatersrand, Johannesburg, South Africa; 4 Department of Family Medicine, Faculty of Health Sciences, University of the Witwatersrand, Johannesburg, South Africa; 5 Department of Family Medicine, Faculty of Medicine, University of Pretoria, Pretoria, South Africa

**Keywords:** family physicians, FCFP (SA) examination, family medicine registrars, postgraduate training, national exit examination, postnatal care

## Abstract

The ‘Mastering your Fellowship’ series provides examples of the question format encountered in the written and clinical examinations for the Fellowship of the College of Family Physicians of South Africa (FCFP [SA]) examination. The series aims to help family medicine registrars prepare for this examination.

## Introduction

This section in the *South African Family Practice Journal* (SAFPJ) is aimed at helping registrars prepare for the Fellowship of the College of Family Physicians of South Africa (FCFP [SA]) examination. It will provide examples of the question formats encountered in the written examination: a multiple choice question (MCQ) in the form of a single best answer (SBA – Type A) or extended matching question (EMQ – Type R), short answer questions (SAQ), questions based on the critical reading of a journal article (CRJ; evidence-based medicine) and an example of an objectively structured clinical examination (OSCE) question. Each question type is presented based on the College of Family Physicians blueprint and the key learning outcomes of the FCFP (SA) programme. The MCQs are based on the 10 clinical domains of family medicine, and the SAQs are aligned with the five national unit standards and the Entrustment Professional Activities (EPAs) based curriculum. The critical reading section will include evidence-based medicine and primary care research methods.

This edition is based on EPA three (Managing women and babies in the postnatal period). We suggest you attempt to answer the questions (by yourself, or with peers, or supervisors) before finding the model answers online: http://www.safpj.co.za/.

Please visit the Colleges of Medicine website for guidelines on the Fellowship examination: https://www.cmsa.co.za/view_exam.aspx?QualificationID=9. We are keen to hear about how this series assists registrars and their supervisors in preparing for the FCFP (SA) examination. Please email us (editor@safpj.co.za) your feedback and suggestions.

## Multiple choice question (MCQ): Single best answer

You are reviewing a baby brought to the clinic for review a few days after delivery. The mom, a 28-year-old human immunodeficiency virus (HIV)-negative woman, P2 G2, had an uneventful delivery at the midwife obstetric unit (MOC). You are asked to review this baby, who had a birth weight of 2.4 kg and is observed to have a nasal discharge. Upon further enquiry, you learn that the mother had dual HIV and syphilis rapid diagnostic tests at 32 weeks gestation. The rapid syphilis test was reactive, and the rapid plasma reagent (RPR) test confirmed the diagnosis. The mom received all three doses of 2.4 MU of benzathine penicillin G intramuscularly (IM) weekly, and the partner was also treated. The last dose was administered 24 days before the delivery of the baby.

What is the most appropriate next step in managing this scenario?

- Admit the baby for intravenous (IV) penicillin.
- Admit the baby for aqueous crystalline penicillin IM.
- Administer benzathine penicillin IM.
- No treatment is necessary for the baby.

Correct answer: (a)

### Discussion

Despite good antenatal attendance and early maternal syphilis testing, there has been a resurgence of congenital syphilis cases in many provinces in South Africa. Adverse pregnancy outcomes occur in up to 80% of syphilis seropositive, untreated pregnant women. About 30% – 40% of babies who acquire syphilis in utero die shortly before or after birth. That has two considerations: the babies’ clinical symptoms and the mother’s treatment status.

All positive rapid tests in pregnant women must be confirmed by a lab-based RPR, which confirms the infections and tells one whether the infection is current and active or a previous infection. It also gives one a baseline titre to monitor response to treatment. One must not delay the first treatment dose if the rapid test is positive and there is no need to wait for the RPR result before starting treatment to avoid delays.

When sending the RPR to the lab, one must send a stand-alone blood sample and request both RPR and a specific syphilis test. If the laboratory has been informed that the rapid test was positive, the laboratory will automatically perform a specific syphilis test to confirm whether or not the rapid test was a false positive. However, low RPR titres can be challenging to know whether there is active, clinically relevant syphilis, which is why the specific syphilis test should be requested from the laboratory if the RPR titre is 1:4 or lower. This will guide the clinician to determine whether or not the client needs to be treated (if the specific test is reactive).

A low RPR titre could also suggest very early syphilis infection. If this is the case, subsequent testing will detect the infection in the following tests, resulting in a positive rapid test and RPR with a higher titre.

In resource-constrained countries, treatment of congenital syphilis is based on the identification of maternal syphilis during the antenatal period or delivery time, RPR results in the infant and whether the seroconverted infant is born with features of congenital syphilis. Also, it is crucial to determine whether the mother received three doses (given on three consecutive weeks) of benzathine penicillin IM and was adequately treated for syphilis, with the last dose being 1 month (30 days) before delivery of the baby.

Inadequately treated mother is defined as:

- the mother did not complete three doses in full; or
- the mother received three doses, but there was a delay of > 14 days between weekly IM doses; or
- the last dose was less than 30 days before delivery, treatment must be completed 30 or more days before delivery for syphilis to be adequately cleared; or
- the dose that the mother received was incorrect.

An untreated mother is when:

- The mother did not receive any treatment for syphilis.
- The mother was treated for syphilis with an antibiotic that was not penicillin. Other antibiotics have been used as alternatives in different populations, but only benzathine penicillin G is effective for preventing congenital syphilis. Mothers with penicillin allergy must be referred to the hospital for desensitisation.

Management of Syphilis-exposed babies with symptomatic syphilis. These babies must have a confirmatory syphilis test (RPR) and ideally send the placenta for histology:

- All babies require admission for parenteral penicillin (Penicillin G).
- If unable to be admitted at the current level of care, refer all babies with suspected congenital syphilis infection to the appropriate level of care for inpatient admission and work-up.
- Refer all symptomatic babies with complications, for example, thrombocytopenia, anaemia, respiratory distress, signs of liver dysfunction and suspected meningitis, to a centre with high care or intensive care unit.
- These babies must be managed at an appropriate level of care because they are seriously ill and have high morbidity and mortality.
- Ten days of aqueous crystalline Penicillin G IV/IM must be given without interruption or missing doses; otherwise, one needs to restart the 10-day course. The IM injections should be in the anterolateral thigh.
- Remember that confirmed congenital syphilis is a notifiable condition.

Management of syphilis-exposed babies with asymptomatic syphilis:

- Single dose of benzathine penicillin G 50 000 units/kg IM must be given and never give it IV.
- Ensure the mother’s partner is traced, tested and treated.

**Further rea ding**

- ART clinical guidelines for the management of HIV in adults, pregnancy, breastfeeding, adolescents, children, infants and neonates. National Department of Health; 2023. Pretoria.
- Peters RP, Nel JS, Sadiq E, et al. Southern African HIV clinicians society guideline for the clinical management of syphilis. South Afr J HIV Med. 2024;25(1):1577. https://doi.org/10.4102/sajhivmed.v25i1.1577
- Congenital syphilis – Frequently asked questions [homepage on the Internet]. National Institute for Communicable Diseases (NICD); 2023 [cited 2025 Feb 17]. Available from: https://www.nicd.ac.za/wp-content/uploads/2023/11/Congenital-Syphilis-FAQ_20231011-2.pdf

## Short answer question (SAQ): Entrustment Professional Activity 22: Ethical and legal practice

You are working in a community health centre. A clinical nurse practitioner from the Integrated Management of Childhood Illness (IMCI) clinic refers a 20-month-old baby boy. He has had a cough, fever and runny nose for the last 3 weeks. The mom visited the clinic 3 days ago and was told it was a viral infection. It is now getting worse; the child has bouts of coughing and then seems to gasp for air. There have been three spells of ‘turning blue’ after such episodes. The child also vomits and appears to be losing weight. The mother is worried about her child, but she is also concerned as she has just had another baby, who is 4-weeks-old, and, at home.

1. **You review the child’s Road to Health Card (RTHC) as part of your assessment. What information would you like to obtain from the RTHC to assist with your assessment of the child’s growth and current nutritional status? (Any 6 from the list below) (6 marks)**
  - Birth history, birth weight and history of prematurity.
  - Maternal health or well-being and birth spacing.
  - Growth trends over time on the growth charts – Anthropometric measurements, that is, weight for age, length for age, mid-upper arm circumference (MUAC) and weight for length/height.
  - Deworming and vitamin A supplement.
  - Record of previous illnesses or admissions (recurrent childhood illnesses).
  - Vertical transmission prevention and/or HIV status.
  - Tuberculosis (TB) exposure.
  - Developmental milestones.
  - Immunised.
2. **On further review, the infant’s immunisation schedule was not up to date. You spend some time exploring this with the mom. It turns out she was hesitant to vaccinate the child following the COVID period, as she had heard that vaccines are not safe. She heard that childhood vaccines can cause autism and that drug companies produce unsafe vaccines for financial gain and then make money from treating the side effects of vaccines. As the family physician, how will you approach counselling this mother so as not to alienate her further? List five broad counselling approaches you will use. (Any 5 of the following) (5 marks)**
  - Accept that this may be an emotionally charged conversation with competing views but try to maintain a *therapeutic relationship*. Roll with resistance; communication skills are key.
  - A *guiding approach*, as opposed to an authoritarian one (used during motivational interviewing for behaviour change), may best serve the therapeutic relationship.
  - *Listen* to the mother’s concerns. The perspective of parents/family/patients as immunisation decision-makers is critical. The practitioner’s responsibility is to be empathetic to the rationale underlying vaccine refusers’ decisions.
  - *Give information*, but don’t claim 100% safety. Provide education on vaccine safety that is factual, science-based information on vaccines, addressing myths and fears that contribute to hesitancy. Reassure the patient/parent that vaccines are mostly safe and prevent life-threatening diseases. Provide information on vaccine benefits and adverse reactions.
  - *Discuss the risks* that accompany not vaccinating one’s child and the risk to the greater community. Patient refusal is often based on inaccurate information or a lack of understanding of the safety and efficacy of vaccines, as well as several myths. Families who refuse or resist vaccination for their children often defend their position regarding what they believe is in the best interests of their children. Address their concerns and respect their autonomy while communicating the immediate risk of not vaccinating the child or the new baby in the family and the greater good of vaccinations in the community.
  - Craft the *middle ground* and acknowledge the position held by the parents and the belief that ongoing engagement is better than disrupting or altogether severing the therapeutic relationship through dismissal. You will ultimately need to respect the wishes of the parent/s.
  - *Information transmission*: Time may be an issue for counselling during the consultation. You may direct the mother towards resources or literature that is more balanced and evidence based. Seek to empower the patient/parent.
  - *Follow-up*: Each encounter should be used as an opportunity to discuss the importance of vaccination.
3. **You refer the child for admission to the hospital as he has signs of pneumonia and respiratory distress. A few days later, you hear he was diagnosed with whooping cough and died during the admission. Vaccine hesitancy was identified by the World Health Organization as one of the top 10 global health threats of 2019. You are disturbed by this case and also have concerns for the other unvaccinated neonate at home. What ethical principles apply in this case? Name and describe four. (8 marks)**
  - *Autonomy*: Informed consent, right of refusal and decision-making capacity are considered. The parent has a right to refuse immunisation for their child and avoid the risk of adverse effects from immunisation. A mother can make informed decisions regarding her child’s health care. However, autonomy is not absolute when it may cause harm to others, such as when an unvaccinated child is at risk of infection and spreading disease. Parents also have a duty of care to their children.
  - *Distributive justice*: Considering the common good by mandating vaccines and herd immunity, and promoting benefits in public health. Benefits and burdens are allocated to those who vaccinate and those who don’t. Who bears the burden of vaccination, and who benefits from herd immunity?
  - *Beneficence*: Vaccinate to help protect those most at risk or not fully vaccinated or protected (young infants, elderly, immunocompromised). Prevent disease in oneself and others. Vaccination debates are similar to other types of decisions that constitute the unspoken social contract – membership in a community often places citizens in the position of supporting actions or policies judged to be for the overall benefit of society, but that might contradict individual beliefs about what is in the best interests of a particular person.
  - *Non-maleficence*: Providers have a responsibility to do no harm. Hesitancy is rooted in fears of vaccine safety, efficacy and long-term profile of vaccines, delayed acceptance or refusal of vaccination, despite the availability of vaccine services on the part of the parent.
4. **Are there legal implications for non-vaccinators in South Africa? (1 mark)**
  No. Regarding the law, South African law does not hold non-vaccinators accountable and legally liable, and there is no retributive justice.
5. **In reference to Q4 above, briefly explain two implications for non-vaccination of children in South Africa. (Any 2 options) (2 marks)**
  - South Africa does not have a law that mandates vaccination, but the *Children’s Act* (*2005*) prioritises the *child’s best interests* over parental choice. If a child’s health is at serious risk because of lack of vaccination, health care providers or authorities may intervene.
  - *School policies* may involve measures such as rejecting enrolment, although they cannot technically discriminate.
  - During disease outbreaks, public health authorities can implement measures such as *exclusion from schools* or quarantines to prevent the spread of infection.
  - If a parent’s refusal to vaccinate leads to *severe harm or death*, they could face *legal consequences* under *child neglect or endangerment laws*.
6. **How would you advise the mother regarding her newborn baby at home? (Any 3 options) (3 marks)**
  - *Urgent vaccination*: If the neonate is asymptomatic with no fever or cough, encourage the parent to follow the immunisation schedule to ensure the newborn receives all recommended vaccines, especially the *DTaP* (*Diphtheria, Tetanus and Pertussis*) *vaccine* at 6 weeks, 10 weeks and 14 weeks, with a booster at 18 months.
  - *Immediate protection*: To reduce the risk of transmission, booster immunisation against pertussis for those in close contact (parents, siblings, caregivers) may be considered.
  - Cough and sneeze *hygiene,* and wash hands with soap and water often.
  - *Early medical attention:* Advise her to *seek immediate care* if the newborn shows signs of illness, such as coughing, feeding difficulties or respiratory distress.

**Total: 25 marks**

**Furth er reading**

- Moodley K. Medical ethics, law and human rights: A South African perspective. Van Schaik Publishers; 2017. Pretoria.
- World Health Organization. Pertussis [homepage on the Internet]. Geneva; 2025 [cited 2025 Feb 18]. Available from: https://www.who.int/health-topics/pertussis#tab=tab_1
- Mathebula L, Cooper S, Zunza M, Wiysonge CS. Assessing routine childhood vaccination acceptance, hesitancy and refusal in Cape Town, Western Cape, South Africa: A mixed-method study protocol. BMJ Open. 2025;15(2):e093451. https://doi.org/10.1136/bmjopen-2024-093451
- Hendrix KS, Sturm LA, Zimet GD, Meslin EM. Ethics and childhood vaccination policy in the United States. Am J Public Health. 2016;106(2):273–278. https://doi.org/10.2105/AJPH.2015.302952

## Critical appraisal of research

Read the accompanying article carefully and then answer the following questions. As far as possible, use your own words. Do not copy out chunks from the article. Be guided by the allocation of marks concerning the length of your responses.

- Mokwena K, Modjadji P. A comparative study of postnatal depression and associated factors in Gauteng and Free State provinces, South Africa. Afr J Prim Health Care Fam Med. 2022;14(1):a3031. https://doi.org/10.4102/phcfm.v14i1.3031

**Total: 30 marks**

1. Did the study address a focussed issue? (4 marks)
2. Critically review how the authors approached the sampling process in this study. (2 marks)
3. Critically review the size of the final sample included in the study. (3 marks)
4. Critically review the description of criteria for inclusion and exclusion in the sample. (5 marks)
5. Critically review how participants were recruited and consented to this study. (3 marks)
6. Evaluate the authors’ methodology for validating and translating their data collection tool. (5 marks)
7. Critically review the description of the members of the research team. (2 marks)
8. Critically review the authors’ approach to managing missing data in the data set. (2 marks)
9. How valuable are the study findings and recommendations to the South African context? (4 marks)

### Suggested answers

1. **Did the study address a focussed issue? (4 marks)**
  - A question can be ‘focussed’ regarding the population studied, the risk factors studied, whether the study tried to detect a beneficial or harmful effect, and the outcomes considered.
  - The PICO (Population, Interventions, Comparators and Outcomes) framework could be used to assess whether the issue studied is focussed. However, a PICO framework is helpful for an experimental study, which is not the design of this study. Given the study’s observational nature, a PECO (Population or Problem, Exposure, Comparison or Control, and Outcome) format is applicable in this study design.
  - The researchers were interested in describing the prevalence of postnatal depression (PND) and comparing two different community settings in terms of prevalence and associated factors.
  - Yes, the researchers aimed to address a focussed issue, to detect the difference in the prevalence of PND (outcome) in women (population) who live in different South African communities (comparison). In addition, it provided factors associated with PND that may explain different contributing variables or risk factors between the two groups (exposures).
2. **Critically review how the authors approached the sampling process in this study. (2 marks)**
  - The researchers obtained the sample of women in a couple of steps. Firstly, they randomly selected facilities and district municipalities. Secondly, the researchers purposively sampled the women in these facilities.
  - The researchers admitted that obtaining a random sample of women waiting in the queues was impossible, therefore they used convenience sampling. This approach limits the interpretation of the study findings. The authors acknowledge that this sampling technique could introduce bias and limit the ability to draw inferences about this population.
3. **Critically review the size of the final sample included in the study. (3 marks)**
  - The researchers calculated a sample size of 344 participants, considering a 5% margin of error and a 95% confidence interval.
  - However, the study included a final sample size of 477 with a split of 240:237 between the two district municipalities.
  - In the ‘limitations’ section of the paper, the authors acknowledged a ‘slight variation of a sample size’, stating that they wanted to strengthen the power of the study and reduce the margin of error. They mention the issue of being unable to remove participants with missing data of less than 10% but did not state during which phase of the study they decided to increase the sample size beyond the calculated number. It was unclear whether the complete-case analysis was done during the recruitment and collection phase or after the completion of the data collection. More information is needed to justify the approach to oversampling.
4. **Critically review the description of criteria for inclusion and exclusion in the sample. (5 marks)**
  - Women who met the inclusion criteria were enrolled in the study based on their availability and accessibility at the time of the study. These included 18-year-old mothers with live births attending health care facilities in two districts who had consented to the study and under-18-year-old mothers who attended the same health care facilities whose guardians assented to the study.
  - The authors provided no exclusion criteria, as only the inclusion criteria were mentioned at the start of the ‘research methods and design’ section: women who delivered a live infant within 12 weeks of data collection and who attended postnatal care at the included facilities.
  - The ethical considerations section mentions that underaged women with no access to caregivers for consent were excluded. Women with existing mental health conditions should also have been excluded, as this study was about screening for PND in a healthy postnatal population.
  - One should also have considered excluding patients who required high-risk review or had other medical comorbidities to have been excluded or would have required capturing these potential contributing factors to PND. The survey only mentioned sociodemographic, obstetric and baby characteristics as contributing factors.
  - In the ethical consideration section, it is stated that research assistants were ready to stop the interview and refer those who expressed emotional distress for mental health support as part of a distress protocol. However, it is unclear how many participants interrupted interviews or required interview termination and referral for support.
5. **Critically review how participants were recruited and consented to this study. (3 marks)**
  - Under ‘data collection’, it states that ‘mothers were individually recruited as they were waiting to be attended to by health professionals for their postnatal check-up and well-baby clinic’.
  - It is implied but not mentioned explicitly that research assistants did this screening for possible recruitment in the waiting areas.
  - After agreeing to participate, participants were given detailed information about the study’s purpose and value, and how the results would be used. It is unknown whether a private room was available for screening, obtaining consent and collecting data.
6. **Evaluate the authors’ methodology for validating and translating their data collection tool. (5 marks)**
  - The data collection tool was validated by Using the Edinburgh Postnatal Depression Scale (EPDS), a reliable and standardised tool for screening PND. This tool was validated previously in South Africa and used in several studies in the context of South Africa.
  - The authors performed additional validation procedures through content and face validity in this study and conducted a pilot study to confirm that the questionnaire effectively measured the intended constructs.
  - Given the previous work done in South Africa, it is unclear why the authors conducted these repeat measures for their study. Furthermore, the authors stated that experts reviewed content and face validity but did not specify these experts’ professional backgrounds and expertise.
  - The authors conducted a pilot study with 15 mothers at a facility that was not part of the main study. The data from this pilot study were not included in the data analysis, which is an appropriate practice. However, given the previous validation work done in past studies, the authors did not justify the time and resources allocated to involving the pilot sample.
  - Using independent translators fluent in IsiZulu, Setswana and English, they performed forward and backward translations of the questionnaire. However, whether a translation was performed during previous studies in South Africa is unclear, as these studies appeared to have been conducted in the same provinces.
7. **Critically review the description of the members of the research team. (2 marks)**
  - Describe the credentials and experience of the research team as part of standard reporting practice. However, the credentials and experience of team members in this study have not been clearly outlined.
  - This study did not describe the roles and contributions of the research and fieldwork team. It is unclear who managed the sampling, recruitment, consenting, data collection and analysis steps.
8. **Critically review the authors’ approach to managing missing data in the dataset. (2 marks)**
  - Missing data may be at random or not at random. Ignoring missing data in statistical analysis can generate severely biased study results. Complete-case analysis (CCA) is one commonly used approach in which all persons with missing values on one or more variables are excluded from the analysis.
  - In this study, the researchers applied a variation of CCA by excluding three questionnaires with more than 10% missing data. It is unclear why this cutoff of 10% was used. Usually, a missing data percentage of 5% is mentioned as a cutoff. However, it should be realised that the rate of missing data and the strength of the relationship between missing and observed variables are important.
9. **How valuable are the study findings and recommendations to the South African context? (4 marks)**
  - The authors use the variable prevalence of PND (22% in this study compared to other recent studies) to advocate for context-specific interventions tailored to higher-risk groups, such as those who have experienced stressful events.
  - The cross-sectional nature and the use of non-probability sampling in the study limit the generalisability of the findings to the broader South African context.
  - The authors recommended routine universal screening for PND in primary health care, which will help mothers get early support and access to basic mental health services. However, the service implications must be reviewed, given the staff shortages in postnatal care and well-baby clinics.
  - Individuals screening positive for PND should be referred to specialised mental health services for accurate diagnosis and care, impacting overall health care coordination.

### Further reading

- Pather M. Evidence-based family medicine. In Mash B, editor. Handbook of family medicine. 4th ed. Cape Town: Oxford University Press, 2017; p. 430–453.
- The Critical Appraisals Skills Programme (CASP). CASP cross-sectional study checklist [homepage on the Internet]. 2025 [cited 2025 Jan 27]. Available from: https://casp-uk.net/casp-tools-checklists/
- JBI. Critical appraisal tools – Checklist for analytical cross-sectional studies [homepage on the Internet]. 2025 [cited 2025 Jan 27]. Available from: https://jbi.global/critical-appraisal-tools
- Sterrantino AF. Observational studies: Practical tips for avoiding common statistical pitfalls. Lancet Reg Health-Southeast Asia. 2024;25:100415. https://doi.org/10.1016/j.lansea.2024.100415
- Heymans MW, Twisk JW. Handling missing data in clinical research. J Clin Epidemiol. 2022;151:185–188. https://doi.org/10.1016/j.jclinepi.2022.08.016

## Objectively structured clinical examination station (OSCE): Entrustment Professional Activity 3: Postpartum contraception

*Objective of station:* This station tests the candidate’s ability to consult with a postpartum patient and advise the patient on contraception.

*Type of station:* Integrated consultation.

*Role player:* A 30-year-old female.

### Instructions for candidate

- You are the family physician working in the postnatal ward of a large district hospital.
- A 30-year-old woman has given birth to her third child via emergency caesarean section 48 h ago. She has a history of gestational diabetes, a strong family history of blood clots and has concerns about postpartum contraception. She is breastfeeding but has read online that contraception can affect the milk supply. She is also worried about weight gain and long-term health effects.
- Your task is to consult with this patient and provide appropriate counselling and advice.
- You do not need to examine this patient. All relevant examination findings will be provided.

### Instructions for the examiner

- This is an integrated consultation station in which the candidate has 20 min.
- Familiarise yourself with the assessor guidelines, which detail the required responses expected from the candidate.
- No marks are allocated. In the mark sheet (Table 1), tick off one of the three responses for each of the competencies listed. Ensure you are clear on the criteria for judging a candidate’s competence in each area.

**TABLE 1:** Marking sheet for consultation station.

Competencies	Candidate’s rating		
	Not competent	Competent	Good
Establishes and maintains a good clinician-patient relationship			
Gathering information: history/examination/investigations			
Clinical reasoning			
Explanation and planning			
Management			

### Guidance for examiners

- The station challenges the candidate to navigate medical complexity, risk assessment, patient hesitancy and an ethical dilemma while ensuring safe contraception choices in a postpartum patient.
- A working definition of competent performance is when the candidate effectively completes the task within the allotted time in a manner that maintains patient safety, even though the execution may not be efficient and well structured:
  ■ *Not competent*: Patient safety is compromised (including ethically and legally) or the task is not completed.
  ■ *Competent*: The task is completed safely and effectively.
  ■ *Good*: In addition to displaying competence, the task is completed efficiently and empathically using a patient-centred approach.

#### Establishes and maintains a good clinician–patient relationship

The competent candidate is respectful and engages with the patient in a dignified manner (*ascertains reason for the consultation and makes the patient feel comfortable while ensuring the ground for confidentiality is set; gives the patient full attention, summarises and reflects back on what the patient says*).

The good candidate is empathic, compassionate and collaborative, facilitating active participation in key areas of the consultation (*demonstrates cultural sensitivity, encourages open conversation while respecting the patient’s autonomy; validates the patient’s concerns; is aware of the husband wanting to be part of this consultation to speak on behalf of the patient*).

#### Gathering information

The competent candidate gathers sufficient information to establish a clinical assessment (*explores past contraceptive use, gestational diabetes,* body mass index (BMI), *clotting risk, menstrual history, previous pregnancies, delivery, complications, cultural beliefs, concerns and fears).*

The good candidate additionally has a structured and holistic approach (*explores partner dynamics, decision-making ability, patient is reluctant to contradict her husband).*

#### Clinical reasoning

The competent candidate identifies the issue at hand and outlines a 3-stage assessment. (*Clinical: postpartum patient with gestational diabetes and increased BMI who has a clotting risk seeks advice for contraception while breastfeeding*; *Individual: Concerned about weight gain, fear of husband, confused, especially after trying to find information on the Internet, and has misconceptions about contraception; Contextual: does not want to contradict husband who believes that hormonal contraception is ‘dangerous’ and demands natural methods only).*

The good candidate additionally recognises that the patient’s initial choice of combined oral contraceptive will increase the risk of clotting disorders because of the recent caesarean section, history of gestational diabetes and strong family history of clotting disorders (< 6 weeks postpartum). In addition, the good candidate excludes migraines.

#### Explaining and planning

The competent candidate uses clear language to explain to the patient the contraception options; discusses the effectiveness, side effects and how each method aligns with breastfeeding; and uses shared decision-making to empower the patient’s choice. Elicits patient’s preferences while confirming understanding.

The good candidate additionally addresses misconceptions, for example, weight gain with hormonal contraception. Addresses the patient’s dilemma – she wants contraception but is afraid of her husband’s reaction.

### Management

The competent candidate outlines safe options while encouraging shared decision-making in developing a personalised contraception plan with the patient.

*Progesterone-only contraceptives – progesterone only pill (POP), injection, implant, hormonal intrauterine contraceptive device (IUCD) – safe for breastfeeding and preferred in women with risk factors for clotting disorders*

*Copper IUCD – no hormones, long-term, but may cause heavier periods*

*Barrier methods – less effective but suitable for spacing pregnancies*

*Permanent methods – tubal ligation – if the family is complete*

The good candidate will sensitively navigate the ethical dilemma, ensuring that the patient is the decision maker while addressing cultural concerns. Additionally, a good candidate supports patient autonomy while managing the husband’s influence diplomatically and provides written material to reinforce evidence-based choices.

### Role play – Instructions for actors (simulated patient and husband)

#### Patient profile

- Name: Mrs. AB
- Age: A 30-year-old woman
- Language: Fluent in English but occasionally defers to her husband in conversation
- Occupation: Housewife
- Marital status: Married for 8 years
- Children: Three (a newborn, a 3-year-old and a 6-year-old)
- Religion and/or cultural background: Hindu, strong family influence on health decisions

#### Medical history

- Pregnancies:
  ■ G3P3 (three pregnancies, three live births, no previous miscarriages)
  ■ First, second child: Normal vaginal delivery
  ■ Third child: Emergency C-section because of foetal distress (48 h ago)
- Gestational diabetes (GDM): Diagnosed during her third pregnancy, resolved postpartum, but at increased risk for type 2 diabetes.
- Baby weight: 3.8 kg; baby well with no hypo/hyperglycaemia
- Family history:
  ■ Strong family history of blood clots (maternal uncle had deep vein thrombosis, grandmother had a stroke).
  ■ Several female relatives avoid hormonal contraception because of cultural beliefs.
- Lifestyle:
  ■ Weight: BMI 32 (Obese)
  ■ Smoking: Non-smoker
  ■ Alcohol: Does not consume alcohol
  ■ Exercise: Minimal activity
  ■ Diet: Traditional Indian diet, high in carbohydrates

#### Current situation and concerns

- Delivery and recovery:
  ■ Delivered by emergency C-section 2 days ago.
  ■ No immediate complications, but experiencing mild pain at the incision site.
- Breastfeeding:
  ■ Exclusively breastfeeding but worried about milk supply.
  ■ Has heard conflicting advice about hormonal contraception reducing milk production.
- Contraception history and preferences:
  ■ Previously used condoms but found them inconvenient.
  ■ Never used hormonal contraception because of family beliefs.
  ■ Read about combined oral contraceptives and want to try.
  ■ Wants long-term contraception but is worried about side effects.
  ■ Has misconceptions about weight gain with contraception.
  ■ Husband strongly opposes hormonal methods and prefers natural methods.

Husband’s role (ethical dilemma component):

- Wants to be part of the consultation to speak for the patient.
- Dominating personality.
- Strongly believes hormonal contraception is dangerous.
- Prefers natural family planning methods.
- Influential in family decisions, making patient hesitant to contradict him.

Patient’s hidden concerns (revealed if explored well):

- Conflicted: She wants effective contraception but doesn’t want to go against her husband’s wishes.
- Fear of conflict: Concerned that choosing contraception could create tension in the marriage.
- Feels pressured: Has seen female relatives in her family avoid contraception because of cultural expectations.

Examiner’s guidance for simulated patient:

- If the candidate asks open-ended questions:
  ■ Express hesitation and look towards the door where the husband is waiting.
  ■ Eventually, reveal that you want long-term contraception but are worried about family and cultural expectations. Read about combined pills and wants to try as they would help reduce her weight.
- If the candidate provides reassurance and private options:
  ■ Respond positively and ask if it can be done discreetly.
- If the candidate fails to address your autonomy:
  ■ Ask the candidate to speak to her husband in your absence.
- If the candidate pushes too hard against your husband’s opinion:
  ■ Look uncomfortable and say, ‘I don’t want to argue with him. Maybe I should just wait and see?’

### Clinical findings

General observations:

- Appearance: Comfortable but slightly anxious.
- Consciousness level: Alert and oriented to time, place and person.
- Emotional state: Mildly hesitant; avoids contradicting her husband.
- Temperature: 37.1 °C.
- Blood pressure: 116/74 mmHg.
- Heart rate: 78 beats per minute (bpm).
- Respiratory rate: 16 breaths per min.
- Blood glucose: 6.2 mmol/L.

Abdominal examination (post-C-section):

- Inspection:
  ■ Pfannenstiel (low transverse) incision, well-approximated.
  ■ No signs of infection (no redness, discharge or swelling).
  ■ The uterus is palpable just above the pubic symphysis (expected involution).
- Palpation:
  ■ There is mild tenderness around the incision site but no guarding or rebound tenderness.
  ■ Uterus firm, non-boggy (no signs of postpartum haemorrhage).
  ■ No palpable masses or distension.
- Auscultation:
  ■ Bowel sounds present, normal.

Breast examination (If asked about breastfeeding issues):

- Breasts:
  ■ Soft, no engorgement.
  ■ Nipples intact, no cracking or bleeding.
  ■ No signs of mastitis (no redness, warmth or swelling).
- Milk flow:
  ■ A good latch was reported, but the patient worried about supply reduction with hormonal contraception.

Pelvic examination:

- External genitalia: No perineal trauma (C-section delivery).
  ■ Vaginal bleeding (Lochia):
  ■ Normal lochia rubra (moderate, red bleeding).
  ■ No clots or foul-smelling discharge (no infection).
- Cervix and uterus:
  ■ No cervical motion tenderness (no signs of infection or retained products).
  ■ Uterus involuting appropriately.

**Further reading**

- Grandi G, Del Savio MC, Tassi A, Facchinetti F. Postpartum contraception: A matter of guidelines. Int J Gynecol Obstet. 2023;164(1):56–165. https://doi.org/10.1002/ijgo.14928
- World Health Organization (WHO). Medical eligibility criteria for contraceptive use: Fifth edition [homepage on the Internet]. 2015 [cited 2025 Feb 10]. Available from: https://www.who.int/publications/i/item/9789241549158
- Pearlman Shapiro M, Avila K, Levi EE. Breastfeeding and contraception counseling: A qualitative study. BMC Pregnancy Childbirth. 2022:22(1):154. https://doi.org/10.1186/s12884-022-04451-2

